# The influence of maternal psychological distress on the mode of birth and duration of labor: findings from the FinnBrain Birth Cohort Study

**DOI:** 10.1007/s00737-022-01212-0

**Published:** 2022-02-12

**Authors:** Kuuri-Riutta Sanni, Ekholm Eeva, Scheinin M. Noora, Korhonen S. Laura, Karlsson Linnea, Karlsson Hasse

**Affiliations:** 1 Department of Clinical Medicine, Turku Brain and Mind Center, FinnBrain Birth Cohort Study, University of Turku, Lemminkäisenkatu 3a, Building: Teutori, 20014 Turku, Finland; 2 Department of Obstetrics and Gynecology, University of Turku and Turku University Hospital, Turku, Finland; 3 Department of Psychiatry, Turku University Hospital and University of Turku, Turku, Finland; 4 Department of Paediatrics and Adolescent Medicine, University of Turku and Turku University Hospital, Turku, Finland; 5 Centre for Population Health Research, Turku University Hospital and University of Turku, Turku, Finland

**Keywords:** Maternal psychological distress, Fear of childbirth, Mode of birth

## Abstract

Antepartum depression, general anxiety symptoms, and pregnancy-related anxiety have been recognized to affect pregnancy outcomes. Systematic reviews on these associations lack consistent findings, which is why further research is required. We examined the associations between psychological distress, mode of birth, epidural analgesia, and duration of labor. Data from 3619 women with singleton pregnancies, from the population-based FinnBrain Birth Cohort Study were analyzed. Maternal psychological distress was measured during pregnancy at 24 and 34 weeks, using the Pregnancy-Related Anxiety Questionnaire-Revised 2 (PRAQ-R2) and its subscale “Fear of Giving Birth” (FOC), the anxiety subscale of the Symptom Checklist-90 (SCL-90) and the Edinburgh Postnatal Depression Scale (EPDS). Mode of birth, epidural analgesia, and labor duration were obtained from the Finnish Medical Birth Register. Maternal psychological distress, when captured with PRAQ-R2, FOC, and SCL-90, increased the likelihood of women having an elective cesarean section (*OR*: 1.04, 95% *CI* 1.01–1.06, *p* = .003; *OR*: 1.13, 95% *CI* 1.07–1.20, *p* < .001; *OR*: 1.06, 95% *CI* 1.03–1.10, *p* = .001), but no association was detected for instrumental delivery or emergency cesarean section. A rise in both the PRAQ-R2, and FOC measurements increased the likelihood of an epidural analgesia (*OR*: 1.02, 95% *CI* 1.01–1.03, *p* = .003; *OR*: 1.09, 95% *CI* 1.05–1.12, *p* < .001) and predicted longer second stage of labor (*OR*: 1.01, 95% *CI* 1.00–1.01, *p* = .023; *OR*: 1.03, 95% *CI* 1.02–1.05, *p* < .001). EPDS did not predict any of the analyzed outcomes. The results indicate that maternal anxiety symptoms (measured using PRAQ-R2, FOC, and SCL-90) are associated with elective cesarean section. Psychological distress increases the use of epidural analgesia, but is not associated with complicated vaginal birth.

**Supplementary Information:**

The online version contains supplementary material available at 10.1007/s00737-022-01212-0.

## Introduction

Psychiatric disorders are the leading cause of disease burden in women from 15 to 44 years (“WHO | The Global Burden of Disease: 2004 Update,” 2014). Prevalence of antenatal depression has been reported to be 7–13% and anxiety even more common, with a prevalence of 15–23% (Bennett et al. 2004; Sinesi et al. 2019). Importantly, antepartum depression, general anxiety, and pregnancy-related anxiety have been recognized as affecting pregnancy outcomes (Field et al. 2010; Grigoriadis et al. 2018; Jarde et al. 2016; Reck et al. 2013).

Symptoms of anxiety or depression in pregnancy have been associated with a higher fear of childbirth (FOC) and a lower pain threshold, which further associate with higher rates of cesarean deliveries and increased use of epidural analgesia during labor (Dencker et al. 2019; Rouhe et al. 2011; Ryding et al. 2015; Saisto et al. 2001). Some studies have found that antenatal depression and FOC, in contrast to antenatal general anxiety, increase the risk of cesarean section (Bayrampour et al. 2015; Laursen et al. 2009; Räisänen et al. 2014; Ryding et al. 2015). Systematic reviews on these associations lack consistent findings (Dencker et al. 2019; Grigoriadis et al. 2018). Acute cesarean section (ACs) as well as elective cesarean section (ElCs) heighten the risk of maternal and fetal complications (Yang & Sun 2017). Nevertheless, the rates of cesarean sections have increased over the past decades in developed countries, partly due to FOC (Ryding et al. 2015; Saisto and Halmesmäki 2003).

FOC has been estimated to prolong labor by an average of 40–47 min and has been associated with an increased risk of dystocia and cesarean section (Adams et al. 2012; Laursen et al. 2009; Ryding et al. 1998; Sydsjö et al. 2013; Waldenström et al. 2006). Antenatal anxiety has been found to increase plasma concentrations of catecholamines that associate with enervated uterine contractility and are consequently suspected of prolonging active labor (Johnson and Slade 2003; Lederman et al. 1978). Nulliparity and use of epidural analgesia have also been associated with prolonged labor, although the influence of epidural analgesia on labor duration is controversial (Halpern and Abdallah 2010; Liao et al. 2005).

The FinnBrain Birth Cohort Study was established to study prenatal stress and its potential effects on child health and development (Karlsson et al. 2018). The current study focuses on pregnancy and birth outcomes and the factors influencing them. More specifically, the aim of the present study was to explore the associations between maternal psychological distress (pregnancy-related anxiety, FOC, general anxiety symptoms, and depressive symptoms) and the mode of birth and the duration of labor; these associations are important as the effects of psychological distress with its various presentations on birth remain controversial. Identifying psychosocial factors that potentially complicate birth is highly relevant for both research and clinical purposes. Several of the previous studies have assessed the effects of depression and anxiety symptoms on pregnancy outcomes separately. However, although often appearing together, depression and anxiety are to some extent distinct conditions, with differential symptom course trajectories and pathophysiology (Lemche et al. 2016; Penninx et al. 2011). Thus, their influence on pregnancy and labor outcomes may vary. Finally, pregnancy-related anxiety is a condition largely distinct from both depression and general anxiety that has important reported associations with maternal and offspring outcomes; therefore, it was felt necessary to investigate this scale together with the other psychological symptoms (Dencker et al. 2019; Grigoriadis et al. 2018; Reck et al. 2013). Previous studies in the present cohort show that variance in the presentation of psychological distress symptoms exist at different times throughout pregnancy (Korja et al. 2018). Subsequently, questionnaire data from two pregnancy trimesters were used in the present study in order to evaluate the specific influence of the time when the symptoms occurred on the selected birth outcomes.

## Material and methods

### Study population

Between 2011 and 2015, the FinnBrain Birth Cohort Study (www.finnbrain.fi) recruited 3808 women in Southwestern Finland at gestational week (gwk) 12. The inclusion criteria were a sufficient knowledge of either Finnish or Swedish and a written informed consent. The participation rate was 66% of those who were informed about the study. Women with twin pregnancies (0.8%) and women who did not provide consent to access their data in the Finnish Medical Birth Register (FMBR) administered by the National Institute of Health and Welfare (5%) were excluded, leaving a total of 3619 pregnancies.

### Procedure and ethical approval

Women were initially approached to participate by a research nurse while they were attending appointments. The Ethics Committee of the Hospital District of Southwest Finland approved the protocol. Research questionnaires were either mailed to the participants or could be completed online. The time points for the assessments were 14, 24, and 34 gwks. The Pregnancy-Related Anxiety Questionnaire (PRAQ-R2) was only included in gwks 24 and 34, which is why these two time points were used in the present study.

### Main outcome

The main outcomes were the mode of birth, use of epidural analgesia, and duration of labor. Birth mode was classified into six categories; spontaneous vaginal birth, vaginal breech birth, vacuum extraction, elective cesarean section (ElCs), acute cesarean section (ACs), and emergency cesarean section (requiring general anesthesia). Spontaneous vaginal birth, vacuum extraction, ACs, and ElCs were included to analyze the association of psychological distress on both attempted vaginal birth and elective cesarean section. Vaginal breech birth and emergency cesarean section were excluded from these analyses due to small group sizes. To investigate the use of epidural analgesia in attempted vaginal birth, spontaneous vaginal birth, vacuum extraction, and ACs were included in the analyses. The duration of labor was calculated for first stage, as the time from commencement of regular contractions (three uterine contractions per 10 min lasting ≥ 1 min) until the time of cervical dilatation to 10 cm during active labor, and for second stage, as starting from the time of first documented examination of cervical dilatation of 10 cm until the birth of the newborn. The duration of first stage was based on a maternal report on the beginning of regular contractions. This information was assessed from the Finnish Medical Birth Register, where the attending doctor of midwife documents the labor shortly after the birth.

### Measures

Prenatal depressive symptoms were assessed using the Edinburgh Postnatal Depression Scale (EPDS), general anxiety symptoms with the anxiety subscale of the Symptom Checklist-90 (SCL-90), and pregnancy-specific anxiety with the Pregnancy-Related Anxiety Questionnaire-Revised 2 (PRAQ-R2) (Cox et al. 1987; Derogatis et al. 1973; Holi et al. 1998; Huizink et al. 2016). The items on the PRAQ-R2 can be arranged into three subscales and the first subscale (PRAQ-R2 F1), “fear of giving birth” (FOC) (consisting of items 2, 6 and 8), was also examined independently (Huizink et al. 2016). Furthermore, as the study population represents a general population in a high-income country, the questionnaire data lacked constant cut-off values, and to be able to depict any possible influence of subthreshold symptoms, the questionnaire scores were analyzed primarily as continuous parameters (Sinesi et al. 2019).

As a measure of socioeconomic status, the level of education was used and defined as low (< 12 years), medium (12–15 years), and high (15 years of education).

For post-hoc analyses, an EPDS cut-off of ≥ 12 points was used as the threshold for clinically significant depressive symptoms (Cox et al. 1987; Shrestha et al. 2016). Regarding PRAQ-R2, for which no cut-off exists in the literature, and which in the general population is often normally distributed, the subjects were divided into two groups based on their PRAQ-R2 and PRAQ-R2 F1 scores. The 25% with the highest score functioned as the “anxiety” group and the 75% with a lower score as the control group. For the total PRAQ-R2, the cut-off value was ≥ 27 points at both time points (gwks 24 and 34) and for the PRAQ-R2 F1 (FOC), the cut-off value was ≥ 8.0 points at gwk 24 and ≥ 9.0 points at gwk 34. The cut-off values were determined using all the questionnaire data available at each time point.

For sensitivity analysis, pregnancy complications were dichotomized as a covariate (0 = no, 1 = yes, any); including diagnosis of proteinuria, pregnancy-induced hypertension, pre-eclampsia, gestational diabetes, and maternal diseases complicating the pregnancy/labor/postpartum.

### Covariates

Maternal age (years), parity (nulliparous/multiparous), and pre-pregnancy body mass index (kg/m^2^) (BMI) were chosen as covariates as they are known to affect the duration of labor and mode of birth (Gunnarsson et al. 2017; Halpern et al. 1998; Treacy et al. 2006; Vahratian et al. 2004). Epidural analgesia (yes/no) was added as a covariate when evaluating labor duration (Halpern et al. 1998; Halpern and Abdallah 2010; Zhang et al. 1999). For sensitivity analyses, induction of labor (yes/no) and maternal pregnancy complications (yes/no) were added to the covariates (Harper et al. 2012).

### Statistical analyses

Data analyses were performed by using the Statistical Package of Social Sciences version 26.0 for Mac (SPSS Inc., Chicago, IL). In descriptive analyses, continuous variables were expressed as means (standard deviations) and categorical variables as percentages. All tests were assessed at a 5% level of significance.

For descriptive statistics, variables from the FMBR were presented to describe the study population, including, e.g., variables concerning the women (age, BMI, parity), labor (induction, onset, episiotomy), and the newborn (birthweight).

The associations between the use of epidural analgesia during labor and maternal psychological distress (PRAQ-R2 and PRAQ-R2 F1; SCL-90; EPDS) were analyzed using logistic regression with epidural analgesia as the dependent variable. Additionally, separate models were used for each scale due to the high correlation between the symptom scales and because of an interest in the potentially differential associations between the different symptom domains and the selected outcomes. The rate of instrumental delivery, ACs and ElCs were determined and the associations between independent and dependent variables were explored using logistic regression.

The associations between psychological distress measures and the durations of the first and second stages of labor were analyzed with a general linear model. The associations between the continuous variables (maternal symptoms and the durations of the first and second stages of labor) were analyzed using separate models for each questionnaire. Labor durations (first and second stages, originally expressed in minutes), used as dependent variables in the models, were transformed using a natural logarithm in order to reduce skewness in their distributions. Furthermore, the point of log transformation was used not only to reduce skewness but to assume multiplicative effects rather than additive effects. The effect sizes (ratios) presented in Table 4 are, however, expressed in the original units, i.e., they are regression coefficients transformed back to minutes, using exponent transformation. A predetermined, fixed set of covariates (maternal age, BMI, and parity) was included in all the models to control for the associations of potential confounders. The models were then repeated by adding epidural analgesia (dichotomous variable; yes/no) as a covariate. Analyses were also conducted excluding preterm deliveries, to evaluate its potential effect on the investigated parameters. For sensitivity analyses, induction of labor (yes/no) and maternal pregnancy complications (yes/no) were added as covariates to the original models.

A post-hoc analysis was performed to assess the potential effects of maternal symptoms using the dichotomized distress scales. Associations in these divided groups were analyzed as described above, and alternatively by substituting the continuous variables with the dichotomous ones.

## Results

### Descriptive statistics

Table 1 presents the characteristics of the study population. Fifty percent of the participants (*n* = 1792) were primiparous. The mean gestational age at birth was 39 + 5 (SD: 12.1) gwks, ranging from 24 + 1 to 42 + 3. Seventy-two percent (*n* = 2599) had a spontaneous vaginal birth, 1% a vaginal breech (*n* = 31), and 11% (*n* = 397) had an assisted vaginal birth (vacuum extraction). Six percent of the women (*n* = 206) was delivered by elective cesarean section and 9% (*n* = 339) by acute cesarean section, making a total of 16% of the deliveries (*n* = 592) by cesarean section. Epidural analgesia was used in 54% of the attempted vaginal deliveries and episiotomy was performed in 8%. Table 2 presents the mean values of the maternal psychological distress measures. The prevalence of marked depressive symptoms (EPDS ≥ 12 points) during pregnancy (gwk 24, 34) was 7.3% (*n* = 2683) (Cox et al. 1987; Shrestha et al. 2016).

**Table 1:** Study characteristics

		Mean (SD)	Range	Percentile (%)	Total (*n* = 3619)
Age		30.2 (4.7)	(17.0–46.0)		3619
BMI^a^		24.7 (4.9)	(15.6–60.6)		3613
Parity	Nulliparous			49.5	1792
	Multiparous			50.5	1827
Education	Low (up to 12 years of education)			30.8	1116
	Medium (13–15 years of education)			24.0	868
	High (over 15 years of education)			27.2	984
Pregnancy complications^b^				17.7	642
Labor onset Pre-term	< 37 gestational weeks	39 + 5 (12.1)	(24 + 1 to 42 + 3)	4.8	179
Labor induction				22.2	804
Amniotomy				37.4	1352
Oxytocin				39.0	1411
Mode of birth	Spontaneous vaginal birth			71.8	2599
	Vaginal breech			0.9	31
	Vacuum extraction			11.0	397
	Elective ceaserean section			5.7	206
	Acute cesarean section			9.4	339
	Emergency emergency cesarean section			1.3	47
Labor duration (minutes)^c^	First stage	444 (288)			2991
	Second stage	31 (28)			3023
	In total	475 (300)			2981
Epidural* (yes)				54.2	1850
Episiotomy*				8.0	242
Birth weight (grams)		3550 (536)	(280–5470)		3619
Apgar*	1 min	8.6 (1.3)			3322
	5 min	9.0 (0.8)			3316

^a^BMI = pre-pregnacy body mass index (kg/m^2^)

^b^Pregnancy complications (yes, any); proteinuria, pregnancy-induced hypertension, pre-eclampsia, gestational diabetes, and maternal diseases complicating the pregnancy/labor/postpartum

^c^Labor duration from all women with statistics written in Finnish Medical Birth Register (includes spontaneous vaginal birth, vaginal breech, vacuum extraction, ACs, and acute emergency cesarean section)

^*^when intended vaginal birth (spontaneous vaginal birth, vacuum extraction, ACs)

**Table 2:** The summary statistics of the symptom scales for maternal depressive, general anxiety, and pregnancy-related anxiety symptoms

	Time point (gwk)	Mean	Range	Response rate (%)^a^
PRAQ-R2	24	23.0	(10–48)	74.1
	34	23.2	(10–50)	69.4
PRAQ-R2 F1	24	6.7	(3–15)	74.1
	34	7.2	(3–15)	69.4
EPDS	24	5.0	(0–25)	74.1
	34	4.9	(0–26)	69.5
SCL-90	24	3.9	(0–33)	74.1
	34	3.2	(0–26)	69.4

^a^Total response rate *n* = 3619

### Epidural analgesia

A one-point increase in the PRAQ-R2 total score at 24 or 34 gwks was associated with a 1.02 times higher likelihood (*OR* 1.02; 95% *CI* = 1.01–1.03, *p* = 0.003 and 95% *CI* = 1.01–1.03, *p* = 0.004) for epidural analgesia. One-point increases in PRAQ-R2 F1 at 24 and 34 gwks were associated with a 1.07 times higher likelihood (95% *CI* = 1.04–1.11, *p* < 0.001) and a 1.10 times higher likelihood (*OR* 1.10; 95% *CI* = 1.06–1.14, *p* < 0.001), for epidural analgesia, respectively. EPDS and SCL-90 scores were not associated with epidural analgesia. (Table 3).

**Table 3:** The associations between maternal psychological distress and epidural analgesia during labor and mode of birth

Psycho-logical symptoms	Time point	Epidural analgesia^a^			Instrumental vaginal delivery^b^			Acute cesarean section^c^			Elective cesarean section^d^		
	gwk	*OR* (95% *CI*)*	*p*-value	*N*	*OR* (95% *CI*)*	*p*-value	*N*	*OR* (95% *CI*)*	*p*-value	*N*	*OR* (95% *CI*)*	*p*-value	*N*
PRAQ-R2	24	1.020 (1.007–1.034)	.003	2457	1.015 (0.996–1.034)	.115	2201	1.004 (0.984–1.025)	.682	2457	1.036 (1.012–1.061)	.003	2615
	34	1.020 (1.006–1.034)	.004	2320	1.009 (0.990–1.028)	.350	2080	1.012 (0.991–1.033)	.258	2320	1.037 (1.012–1.062)	.004	2461
PRAQ-R2 F1	24	1.074 (1.038–1.112)	< .001	2458	1.044 (0.996–1.095)	.075	2202	1.006 (0.955–1.059)	.833	2458	1.138 (1.074–1.206)	< .001	2616
	34	1.097 (1.058–1.137)	< .001	2320	1.009 (0.960–1.061)	.720	2080	1.035 (0.982–1.091)	.204	2320	1.130 (1.062–1.203)	< .001	2461
EPDS	24	0.997 (0.977–1.019)	.801	2458	0.982 (0.951–1.014)	272	2202	1.001 (0.968–1.034)	.961	2458	1.018 (0.980–1.057)	.353	2617
	34	1.011 (0.989–1.034)	.317	2322	0.996 (0.964–1.028)	.791	2080	1.010 (0.976–1.045)	.582	2322	1.037 (0.997–1.078)	.072	2464
SCL-90	24	0.985 (0.965–1.006)	.154	2456	0.978 (0.948–1.009)	.162	2201	0.998 (0.966–1.031)	.917	2456	1.028 (0.992–1.065)	.127	2614
	34	1.010 (0.987–1.034)	.389	2320	1.014 (0.981–1.047)	.415	2079	1.019 (0.984–1.054)	.287	2320	1.064 (1.027–1.103)	.001	2460

^a^Epidural as dichotomous variable (Reference = no epidural)

^b^Instrumental vaginal delivery (Reference = spontaneous vaginal birth)

^c^Acute cesarean section (Reference = spontaneous vaginal birth, vacuum extraction)

^d^Elective cesarean section (Reference = spontaneous vaginal birth, vacuum extraction, and ACs)

^*^The ORs describe the relative changes in the odds for epidural analgesia/adverse mode of birth per one-point increase in each psychological distress scale

Post-hoc analyses (see Table 5, supplementary material) revealed that the women in the highest PRAQ-R2 quartile at 24 and 34 gwks more frequently received epidural analgesia, with ORs of 1.30 (95% *CI* = 1.07–1.59, *p* = 0.008) and 1.29 (95% *CI* = 1.05–1.57, *p* = 0.014), compared to the women in the lowest three quartiles. Furthermore, women in the highest PRAQ-R2 F1 quartile more frequently received epidural analgesia, with ORs of 1.27 (95% *CI* = 1.06–1.53, *p* = 0.012) and 1.36 (95% *CI* = 1.11–1.66, *p* = 0.003), compared to those in the lowest three quartiles of PRAQ-R2 F1. Those scoring ≥ 12 points on the EPDS did not receive epidural analgesia more frequently than those scoring < 12 points. These findings were not sensitive to adding maternal pregnancy complications as a covariate (yes/no).

### Mode of birth

Table 3 presents the adjusted odds ratios (ORs) for instrumental vaginal delivery, acute cesarean section, and elective cesarean section, when excluding emergency cesarean sections and vaginal breech births. A one-point increase in PRAQ-R2 was associated with a 1.04 times higher likelihood (*OR* 1.04; 95% *CI* = 1.01–1.06, *p* = 0.003 and 95% *CI* = 1.01–1.06, *p* = 0.004) of ElCs (24 and 34 gwks). Furthermore, a one-point increase in the PRAQ-R2 F1, the subscale of FOC, was associated with a 1.14 (*OR* 1.14; 95% *CI* = 1.07–1–21, *p* < 0.001) and 1.13 (*OR* 1.13; 95% *CI* = 1.06–1.20, *p* < 0.001) times higher likelihood of ElCs (24 and 34 gwks). For general anxiety measures, a one-point increase in SCL-90 was associated with a 1.06 times higher likelihood (*OR* 1.06; 95% *CI* = 1.03–1.10, *p* = 0.001) of ElCs at 34 gwks, but when measured at 24 gwks, the SCL-90 scores did not predict ElCs (*p* = 0.13). EPDS did not predict ElCs at either time point. Of the selected covariates, parity and BMI did not predict ElCs, whereas maternal age did.

Increases in PRAQ-R2, PRAQ-R2 F1, EPDS, or SCL-90 scores (gwk 24 and 34) were not associated with an increased risk for instrumental vaginal delivery or ACs, when age, BMI, and parity were controlled for. These findings were not sensitive to the exclusion of pre-term deliveries or to adding maternal pregnancy complications as a covariate (yes/no).

### Duration of labor

PRAQ-R2 total score, EPDS, or SCL-90 were not associated with the duration of the first stage of labor when age, BMI, and parity were controlled for. A one-point increase in PRAQ-R2 F1 at gwk 24 was associated with a 1.2% increase (*OR* 1.01; 95% *CI* = 0.2–2.3, *p* = 0.024) in the first stage of labor, but this association was not seen with the PRAQ-R2 F1 at 34 gwks (Table 4; 1). When epidural analgesia was controlled for, this association was no longer significant (Table 4; 2).

**Table 4:** The association between psychological distress and labor duration

		Time point	Duration of First stage of labor (cervical dilatation)*		Total	Duration of Second stage of labor (active pushing)*		Total
		(gwk)	Ratio (95% *CI*)	*p*-value	*N*	Ratio (95% *CI*)	*p*-value	*N*
1^a^	PRAQ-R2	24	1.002 (0.998–1.006)	.363	2173	1.006 (1.001–1.012)	.023	2192
		34	1.000 (0.996–1.005)	.834	2055	1.004 (0.998–1.009)	.187	2074
	PRAQ-R2 F1	24	1.012 (1.002–1.023)	.024	2174	1.033 (1.019–1.047)	< .001	2193
		34	1.007 (0.996–1.018)	.207	2055	1.032 (1.018–1.046)	< .001	2074
	EPDS	24	1.004 (0.997–1.010)	.243	2175	1.005 (0.996–1.013)	.300	2194
		34	1.001 (0.995–1.008)	.682	2056	1.002 (0.993–1.011)	.661	2074
	SCL-90	24	1.000 (0.993–1.006)	.948	2174	1.005 (0.994–1.013)	.261	2193
		34	1.004 (0.997–1.011)	.307	2054	1.006 (0.997–1.015)	.213	2073
2^b^	PRAQ-R2	24	1.000 (0.996–1.004)	.984	2173	1.005 (1.000–1.010)	.056	2192
		34	0.998 (0.994–1.002)	.417	2055	1.003 (0.997–1.008)	.355	2074
	PRAQ-R2 F1	24	1.005 (0.995–1.015)	.342	2174	1.029 (1.015–1.043)	< .001	2193
		34	0.998 (0.988–1.008)	.686	2055	1.027 (1.014–1.041)	< .001	2074
	EPDS	24	1.004 (0.998–1.010)	.203	2175	1.005 (0.996–1.013)	.285	2194
		34	1.000 (0.994–1.006)	.994	2056	1.001 (0.992–1.010)	.807	2074
	SCL-90	24	1.001 (0.995–1.007)	.771	2174	1.006 (0.997–1.014)	.189	2193
		34	1.002 (0.995–1.009)	.537	2054	1.005 (0.996–1.014)	.288	2073

^a^In first model age, BMI, and parity adjusted for

^b^In second model epidural added as covariates as a dichotomous variable (Reference = no epidural)

^*^The ratios describe the relative changes in the duration variables per one-point increase in each psychological distress scale

^*^Spontaneous vaginal birth, vacuum extraction, and ACs included in analysis

Regarding the second stage of labor, a one-point increase in the PRAQ-R2 total score at 24 gwks was associated with a 0.6% increase (*OR* 1.01; 95% *CI* = 0.1–1.2, *p* = 0.023) in the second stage of labor, but no association was present with the gwk 34 PRAQ-R2 total score. When epidural analgesia was controlled for, PRAQ-R2 did not predict a longer second stage of labor. One-point increases in PRAQ-R2 F1, at both gwk 24 and gwk 34, were associated with a 3.3% and 3.2% increase (*OR* 1.03; 95% *CI* = 1.9–4.7, *p* < 0.001 and *OR* 1.03; 95% *CI* = 1.8–4.6, *p* < 0.001) in the second stage of labor. When epidural analgesia was added in the analyses as a covariate, PRAQ-R2 F1 remained a significant predictor of the duration of the second stage of labor. A one-point increase in PRAQ-R2 F1 at gwk 24 and at gwk 34 was associated with an 2.9% and 2.7% increase (*OR* 1.03; 95% *CI* = 1.5–4.3, *p* < 0.001 and *OR* 1.03; 95% *CI* = 1.4–4.1, *p* < 0.001) in the second stage of labor.

EPDS or SCL-90 scores were not associated with the duration of the second stage of labor. Adding labor induction or maternal pregnancy complications (yes/no) as a covariate did not affect the results.

In post-hoc analyses (see Table 6, supplementary material), psychological distress was not associated with the duration of the first stage of labor when the groups were dichotomized. However, the second stage of labor was, on average, 8.4% (*OR* 1.08; 95% *CI* = 0.2–17.5, *p* = 0.046) longer in women in the highest PRAQ-R2 total score quartile at gwk 24 but this association fell short of statistical significance when epidural analgesia was added as a covariate (*OR* 1.07, 95% *CI* =  − 1.3–15.6, *p* = 0.102). The women in the highest quartile of PRAQ-R2 F1 scores at gwk 24 had, on average, 15.1% (*OR* 1.15; 95% *CI* = 6.9–24.1, *p* < 0.001) longer second stages of labor when compared to the control women in the lower three quartiles. Those in the highest quartile of PRAQ-R2 F1 at gwk 34 had, on average, a 13.2% (*OR* 1.13; 95% *CI* = 4.5–22.5, *p* = 0.002) longer second stages of labor. This association persisted after controlling for the effect of epidural analgesia; those in the highest quartile at gwk 24 had, on average, 13,8% (*OR* 1.13; 95% *CI* = 5.7–22.5, *p* = 0.001) and at gwk 34, 11.2% (*OR* 1.11; 95% *CI* = 2.7–20.3, *p* = 0.008) longer second stages of labor, respectively. EPDS scores ≥ 12 points did not predict the duration of the second stage of labor at either time point.

## Discussion

### Main findings

Psychological distress as measured by our parameters was not associated with a higher risk for instrumental delivery or acute cesarean section (ACs); however, pregnancy-related anxiety did increase the odds for elective cesarean section (ElCs). Women, who scored highest in pregnancy-related anxiety (PRAQ-R2) and/or fear of childbirth (FOC), more frequently received epidural analgesia during vaginal birth. PRAQ-R2 and its subscale of FOC were also associated with a longer second stage of labor. However, after controlling for epidural analgesia, only the FOC scale independently predicted a longer second stage of labor.

### Interpretation

Our findings are in line with previous studies, reporting prolonged labor duration and labor dystocia/protracted labor in women with FOC (Adams et al. 2012; Laursen et al. 2009; Sydsjö et al. 2013). In a study by Adams et al. comprising 2206 women, this association was only significant in nulliparous women who received epidural analgesia (Adams et al. 2012). However, our study did not stratify between nulliparous/multiparous women with/without epidural analgesia, but instead controlled for these potential confounders in the statistical analyses, thus increasing the generalizability of the findings. Another study of 990 parous women evaluated secondary FOC determined by interviews (with psychotherapists or obstetricians at a special unit) and found that FOC prolonged active labor by 40-min (Sydsjö et al. 2013). However, these studies measured the total duration of active labor, whereas our study categorized the duration of labor into the opening and the pushing stage (Adams et al. 2012; Sydsjö et al. 2013). Our results are in line with findings of Sydsö et al., but indicate that the prolongation occurs during the second stage. A larger study of 25,297 healthy nulliparous women evaluated uncomplicated term pregnancies and found that the clinical diagnosis of dystocia was more frequent in women with FOC. The study evaluated FOC with a single question (“Are you anxious about the course of the upcoming delivery?”, where the response “Yes, a lot” was interpreted as FOC) (Laursen et al. 2009). Pregnancy-related anxiety, when assessed by PRAQ-R, has previously predicted the duration of labor, as was also seen in our study (Reck et al. 2013).

Pain and worry about maternal and fetal complications during the active pushing stage may impair pushing in women with FOC. Furthermore, FOC has been associated with a lower pain threshold and a poorer ability to cope with stress (Ryding et al. 1998; Saisto et al. 2001). In parous women, FOC often arises from a previous negative birth experience (Dencker et al. 2019; Saisto and Halmesmäki 2003). FOC persists throughout pregnancy and is known to elevate plasma catecholamine levels and may thus interfere with uterine contractility, leading to prolonged labor (Lederman et al. 1978). However, even though FOC also prolonged the second stage of labor in our study, we do not consider the prolongation clinically significant, as a one-point increase in FOC only lengthened the labor, on average, by 1 min. Furthermore, FOC did not increase the risk for adverse birth outcomes such as instrumental delivery or ACs, in contrast to the findings of some previous studies (Laursen et al. 2009; Ryding et al. 1998). As FOC only prolonged the second stage of labor in our study, this suggests that it does not associate with labor progression during the opening stage, which might be one of the fears among the women suffering from FOC. Proper pain and labor management, and taking into account a personalized consideration of psychological distress and fears can successfully support the progression of labor during the opening stage.

Our study did not find psychological distress to associate with obstetric interventions such as instrumental vaginal delivery or ACs. However, pregnancy-related anxiety, FOC and general anxiety symptoms (at 34 gwks) increased the odds of an elective cesarean section. FOC has been reviewed as a risk factor for ACs or cesarean section in general, but increased odds were limited to ElCs in our results (Laursen et al. 2009; Ryding et al. 1998; Sydsjö et al. 2013). In a large study of the Danish National Birth Cohort, researchers excluded all women with medical conditions that could interfere with vaginal birth and found that nulliparous women with a FOC had an increased risk of ACs (Laursen et al. 2009). Accordingly, in a case–control study by Ryding et al. comprising 281 women, those who delivered by ACs had a greater FOC (assessed by W-DEQ) in the third trimester compared to those who delivered vaginally (Ryding et al. 1998). Other smaller studies have not found associations between FOC (measured by W-DEQ or a single question) and operative vaginal delivery or ACs (Heimstad et al. 2006; Johnson & Slade 2002; Waldenström et al. 2006). Most of the studies showing no associations between FOC and ACs have used a validated questionnaire (W-DEQ), as compared to the study by Laursen et al., where high anxiety was interpreted as a FOC (Heimstad et al. 2006; Johnson and Slade 2002; Laursen et al. 2009). Our study indicates that women with maternal psychological distress deliver more frequently by ElCs. Nevertheless, psychological distress, especially FOC, also independently leads to the decision to perform ElCs.

General anxiety and depressive symptoms (assessed by the State-Trait Anxiety Inventory, i.e., STAI and General Health Questionnaire) have seldom been associated with the mode of birth, a result in line with our study (Bayrampour et al. 2015; Perkin et al. 1993; Reck et al. 2013; Ryding et al. 1998; Wu et al. 2002). The contradictory results may partly be attributable to different practices in performing ElCs based on maternal distress and/or requests. In a Canadian study of 2825 women, where the only non-somatic indication for ElCs was maternal anxiety due to previous intrauterine death, depression measured by EPDS in the third trimester doubled the risk for an ACs (Bayrampour et al. 2015). In Finland, FOC is accepted as an indication for performing ElCs. This may exclude women with great distress from attempting a vaginal birth and, thus, mitigate any possible effects of distress on an attempted vaginal birth (Rouhe et al. 2011; Saisto and Halmesmäki 2003). Another study of 959 women found an increase in the use of epidural analgesia and operative deliveries in women with high depression scores (defined as Beck Depression Inventory points > 14.5) measured in the third trimester. However, when epidural analgesia was controlled for, the association regarding operative deliveries was no longer evident. (Chung et al. 2001).

### Strengths and limitations

The strengths of the present study are the large number of pregnant women, the assessment at two time points during pregnancy using standardized validated symptom questionnaires, and a Cronbach’s alphas ranging from 0.833 to 0.839. Pregnancy-related anxiety, FOC, depressive symptoms and general anxiety symptoms were independently analyzed, thus covering a wide range of maternal psychological distress and we were able to link subjective questionnaire data with obstetric data from the national register (Gissler et al. 1998). Our study population represents a general population in a high-income country and marked depressive symptoms (EPDS ≥ 12) were only detected in 7.3% of the women, which could dilute some associations; conversely, the welfare and homogeneity of this sample could also be considered a strength of the study, as residual confounders are likely to be limited (Cox et al. 1987; Karlsson et al. 2018; Shrestha et al. 2016).

## Conclusion

Our results indicate that different psychological distress profiles are liable to influence birth in somewhat different ways. Depressive symptoms or general anxiety symptoms did not influence the duration of birth or the use of epidural analgesia, whereas pregnancy-related anxiety and FOC did, regardless of the time of assessment. Health care providers could consider taking notice of the PRAQ-R2 and its subscale FOC in the late second trimester, since these parameters seem to influence the birth outcomes. However, it was observed that high levels of general anxiety symptoms at 34 gwks were associated with ElCs, whereas at 24 gwks this association was not found, suggesting that presentation of general anxiety becomes significant as labor onset approaches. Therefore, it could be helpful, for example, to ameliorate anxiety by providing sufficient support and information regarding birth. Our results persisted even when pregnancy complications were taken into consideration. Different domains of psychological distress were not observed to associate with a complicated birth when vaginal birth was planned; however, they may have other impacts on women’s psychological well-being in the postnatal period that were not investigated here.

## Supplementary Information

Below is the link to the electronic supplementary material.

Supplementary file1 (DOCX 50 KB)

## Data Availability

The datasets generated for this study will not be made publicly available because of restrictions imposed by the Finnish law and the study’s ethical permissions do not allow sharing of the data used in this study. Requests to access the datasets should be directed to the Principal investigator of the FinnBrain Birth Cohort Study.
