# Supplementary material for: The influence of maternal psychological distress on the mode of birth and duration of labor: findings from the FinnBrain Birth Cohort Study
Source: Arch Womens Ment Health. 2022 Feb 12;25(2):463–72. doi: 10.1007/s00737-022-01212-0 (PMC8921080; doi:10.1007/s00737-022-01212-0)
Supplement: Supplementary file 1 — Supplementary file1 (DOCX 50 KB) [file 737_2022_1212_MOESM1_ESM.docx]

Supporting Information (post hoc)

*Table 5.* The association between maternal psychological distress (divided to dichotomous variables by cut-off values^a^) and epidural analgesia during labor.

|  | Cut-off | Cut-off value (points) | Time point (gwks) | OR (95% CI) | p-value | Total (N) |
| --- | --- | --- | --- | --- | --- | --- |
| PRAQ-R2 | ≥75% | ≥27.0 | 24 | 1.30 (1.07-1.59) | .008 | 2457 |
|  | ≥75% | ≥27.0 | 34 | 1.29 (1.05-1.57) | .014 | 2320 |
| PRAQ-R2 F1 | ≥75% | ≥8.0 | 24 | 1.27 (1.06-1.53) | .012 | 2458 |
|  | ≥75% | ≥9.0 | 34 | 1.36 (1.11-1.66) | .003 | 2320 |
| EPDS |  | ≥12 | 24 | 0.88 (0.63-1.22) | .44 | 2458 |
|  |  | ≥12 | 34 | 1.31 (0.92-1.86) | .13 | 2322 |

*^a^Cut-off values for PRAQ-R2 and PRAQ-R2 F1 was upper 25% quartile (Reference = lower 75%) For EPDS cut-off value was ≥12. (Reference = EPDS <12)*

*Table 6.* Post hoc analyses of maternal symptoms of psychological distress divided to dichotomous variables by cut-off values and labor duration

|  |  | Cut-off | Time point | Cut-off value * | Above cut- off | Total | Duration of First stage of labor |  | Total | Duration of Second stage of labor |  |
| --- | --- | --- | --- | --- | --- | --- | --- | --- | --- | --- | --- |
|  |  |  | gwks | Question-naire  points | % | N | OR (CI 95%) | p-value | N | OR (CI 95%) | p-value |
| 1^a^ | PRAQ-R2 | ≥75% | 24 | ≥27.0 | 27.4 | 2173 | 1.027 (0.967-1.092) | .384 | 2192 | 1.084 (1.002-1.175) | .046 |
|  |  |  | 34 | ≥27.0 | 29.1 | 2055 | 0.986 (0.927-1.048) | .645 | 2074 | 1.041 (0.960-1.127) | .334 |
|  |  |  |  |  |  |  |  |  |  |  |  |
|  | PRAQ-R2 F1 | ≥75% | 24 | ≥8.0 | 33.4 | 2174 | 1.031 (0.974-1.092) | .287 | 2193 | 1.151 (1.069-1.241) | <.001 |
|  |  |  | 34 | ≥9.0 | 28.2 | 2055 | 1.020 (0.960-1.084) | .525 | 2074 | 1.132 (1.045-1.225) | .002 |
|  |  |  |  |  |  |  |  |  |  |  |  |
|  | EPDS |  | 24 | ≥12 | 7.3 | 2175 | 1.004 (0.905-1.114) | .941 | 2194 | 1.063 (0.927-1.218) | .385 |
|  |  |  | 34 | ≥12 | 7.3 | 2056 | 1.012 (0.910-1.126) | .818 | 2074 | 0.979 (0.852-1.124) | .764 |
|  |  |  |  |  |  |  |  |  |  |  |  |
| 2^b^ | PRAQ-R2 | ≥75% | 24 | ≥27.0 | 27.4 | 2173 | 1.000 (0.944-1.059) | .995 | 2192 | 1.068 (0.987-1.156) | .102 |
|  |  |  | 34 | ≥27.0 | 29.1 | 2055 | 0.962 (0.907-1.019) | .189 | 2074 | 1.026 (0.948-1.111) | .522 |
|  |  |  |  |  |  |  |  |  |  |  |  |
|  | PRAQ-R2 F1 | ≥75% | 24 | ≥8.0 | 33.4 | 2174 | 1.007 (0.954-1.063) | .800 | 2193 | 1.138 (1.057-1.225) | .001 |
|  |  |  | 34 | ≥9.0 | 28.2 | 2055 | 0.987 (0.931-1.046) | .655 | 2074 | 1.112 (1.027-1.203) | .008 |
|  |  |  |  |  |  |  |  |  |  |  |  |
|  | EPDS |  | 24 | ≥12 | 7.3 | 2175 | 1.011 (0.917-1.115) | .826 | 2194 | 1.066 (0.931-1.220) | .351 |
|  |  |  | 34 | ≥12 | 7.3 | 2056 | 0.990 (0.896-1.094) | .844 | 2074 | 0.966 (0.842-1.107) | .616 |
|  |  |  |  |  |  |  |  |  |  |  |  |

*^a^ In first model age, BMI and parity adjusted for*

*^b^ In second model epidural added as covariates as a dichotomous variable (Reference = No epidural)*

** Cut-off values for PRAQ-R2 and PRAQ-R2 F1 was upper 25% and lower 75% (Reference = lower 75%). For EPDS cut-off value was ≥12 (Reference = <12 points)*

*Spontaneous vaginal birth, vacuum extraction and ACs included in both analysis*

Table 7. Sensitivity analysis – The associations between maternal psychological distress and epidural analgesia during labor and mode of birth with maternal pregnancy complications (proteinuria, pregnancy-induced hypertension, pre-eclampsia, gestational diabetes and other maternal diseases complicating the pregnancy/labor/postpartum) included as a covariate

| Psycho-logical symptoms | Time point | Epidural analgesia^a^ |  |  | Instrumental vaginal delivery^b^ |  |  | Acute cesarean section^c^ |  |  | Elective cesarean section^d^ |  |  |
| --- | --- | --- | --- | --- | --- | --- | --- | --- | --- | --- | --- | --- | --- |
|  | gwk | OR (95% CI)* | p-value | N | OR (95% CI)* | p-value | N | OR (95% CI)* | p-value | N | OR (95% CI)* | p-value | N |
|  |  |  |  |  |  |  |  |  |  |  |  |  |  |
| PRAQ-R2 | 24 | 1.020 (1.007-1.034) | .003 | 2457 | 1.015 (0.996-1.034) | .118 | 2201 | 1.004 (0.984-1.024) | .712 | 2457 | 1.036 (1.012-1.061) | .003 | 2615 |
|  | 34 | 1.020 (1.006-1.034) | .004 | 2320 | 1.009 (0.990-1.028) | .349 | 2080 | 1.012 (0.991-1.032) | .266 | 2320 | 1.037 (1.012-1.062) | .004 | 2461 |
| PRAQ-R2 F1 | 24 | 1.074 (1.037-1.112) | <.001 | 2458 | 1.043 (0.994-1.094) | .080 | 2202 | 1.004 (0.953-1.057) | .886 | 2458 | 1.139 (1.075-1.207) | <.001 | 2616 |
|  | 34 | 1.097 (1.058-1.137) | <.001 | 2320 | 1.009 (0.960-1.061) | .730 | 2080 | 1.034 (0.981-1.090) | .215 | 2320 | 1.131 (1.063-1.205) | <.001 | 2461 |
| EPDS  SCL-90 | 24  34  24 | 0.997 (0.976-1.018)  1.011 (0.989-1.033)  0.985 (0.965-1.005) | .764  .328  .148 | 2458  2322  2456 | 0.982 (0.951-1.014)  0.995 (0.964-1.028)  0.978 (0.947-1.009) | .261  .778  .157 | 2202  2080  2201 | 1.000 (0.967-1.033)  1.009 (0.975-1.044)  0.998 (0.966-1.031) | .982  .616  .894 | 2458  2322  2456 | 1.019 (0.981-1.058)  1.037 (0.997-1.079)  1.028 (0.992-1.065) | .342  .069  .124 | 2617  2464  2614 |
|  | 34 | 1.010 (0.987-1.033) | .399 | 2320 | 1.013 (0.981-1.046) | .431 | 2079 | 1.018 (0.984-1.054) | .303 | 2320 | 1.065 (1.027-1.103) | .001 | 2460 |

^a^ Epidural as dichotomous variable (Reference = No epidural)

*^b^ Instrumental vaginal delivery (Reference = Spontaneous vaginal birth)*

*^c^ Acute cesarean section (Reference = Spontaneous vaginal birth, Vacuum extraction)*

*^d^ Elective cesarean section (Reference = Spontaneous vaginal birth, Vacuum extraction, ACs)*

** The ORs describe the relative changes in the odds for epidural analgesia/adverse mode of birth per one point increase in each psychological distress scale*

*Table 8.* Sensitivity analysis – The association between psychological distress and labor duration with maternal pregnancy complications (proteinuria, pregnancy-induced hypertension, pre-eclampsia, gestational diabetes and other maternal diseases complicating the pregnancy/labor/postpartum) included as a covariate

| c |  | Time point | Duration of First stage of labor* |  | Total | Duration of Second stage of labor* |  | Total |
| --- | --- | --- | --- | --- | --- | --- | --- | --- |
|  |  | (gwk) | Ratio (95% CI) | p-value | N | Ratio (95% CI) | p-value | N |
| 1^a^ | PRAQ-R2 | 24 | 1.002 (0.998-1.006) | .344 | 2173 | 1.006 (1.001-1.012) | .022 | 2192 |
|  |  | 34 | 1.000 (0.996-1.005) | .835 | 2055 | 1.004 (0.998-1.009) | .187 | 2074 |
|  |  |  |  |  |  |  |  |  |
|  | PRAQ-R2 F1 | 24 | 1.013 (1.002-1.023) | .019 | 2174 | 1.033 (1.019-1.047) | <.001 | 2193 |
|  |  | 34 | 1.007 (0.996-1.018) | .203 | 2055 | 1.032 (1.018-1.046) | <.001 | 2074 |
|  |  |  |  |  |  |  |  |  |
|  | EPDS | 24 | 1.004 (0.998-1.011) | .216 | 2175 | 1.005 (0.996-1.013) | .292 | 2194 |
|  |  | 34 | 1.001 (0.995-1.008) | .668 | 2056 | 1.002 (0.993-1.011) | .658 | 2074 |
|  |  |  |  |  |  |  |  |  |
|  | SCL-90 | 24 | 1.000 (0.994-1.006) | .988 | 2174 | 1.005 (0.994-1.013) | .256 | 2193 |
|  |  | 34 | 1.004 (0.997-1.011) | .299 | 2054 | 1.006 (0.997-1.015) | .212 | 2073 |
|  |  |  |  |  |  |  |  |  |
| 2^b^ | PRAQ-R2 | 24 | 1.000 (0.996-1.004) | .981 | 2173 | 1.005 (1.000-1.011) | .054 | 2192 |
|  |  | 34 | 0.998 (0.994-1.002) | .412 | 2055 | 1.003 (0.997-1.008) | .357 | 2074 |
|  |  |  |  |  |  |  |  |  |
|  | PRAQ-R2 F1 | 24 | 1.005 (0.995-1.015) | .297 | 2174 | 1.029 (1.016-1.043) | <.001 | 2193 |
|  |  | 34 | 0.998 (0.988-1.008) | .694 | 2055 | 1.027 (1.014-1.041) | <.001 | 2074 |
|  |  |  |  |  |  |  |  |  |
|  | EPDS | 24 | 1.004 (0.998-1.010) | .171 | 2175 | 1.005 (0.996-1.013) | .273 | 2194 |
|  |  | 34 | 1.000 (0.994-1.006) | .973 | 2056 | 1.001 (0.992-1.010) | .802 | 2074 |
|  |  |  |  |  |  |  |  |  |
|  | SCL-90 | 24 | 1.001 (0.995-1.007) | .716 | 2174 | 1.006 (0.997-1.014) | .182 | 2193 |
|  |  | 34 | 1.002 (0.996-1.009) | .523 | 2054 | 1.005 (0.996-1.014) | .286 | 2073 |

*^a^ In first model age, BMI and parity adjusted for*

*^b^ In second model epidural added as covariates as a dichotomous variable (Reference = No epidural)*

**The ratios describe the relative changes in the duration variables per one-point increase in each psychological distress scale*

*Spontaneous vaginal birth, vacuum extraction and ACs included in both analysis*

*Table 9.* The associations between maternal psychological distress and epidural analgesia during labor and mode of birth with 3- and 5-point increased OR’s (*significant OR’s bolded)**

| Psycho-logical symptoms | Time point | Epidural analgesia |  |  | Instrumental vaginal delivery |  |  | Acute cesarean section |  |  | Elective cesarean section |  |  |
| --- | --- | --- | --- | --- | --- | --- | --- | --- | --- | --- | --- | --- | --- |
|  | gwk | OR (95%  CI) | OR^3^ | OR^5^ | OR (95% CI) | OR^3^ | OR^5^ | OR (95% CI) | OR^3^ | OR^5^ | OR (95% CI) | OR^3^ | OR^5^ |
|  |  |  |  |  |  |  |  |  |  |  |  |  |  |
| PRAQ-R2 | 24 | **1.020 (1.007-1.034)** | **1.06** | **1.10** | 1.015 (0.996-1.034) | 1.05 | 1.08 | 1.004 (0.984-1.025) | 1.01 | 1.02 | **1.036 (1.012-1.061)** | **1.11** | **1.19** |
|  | 34 | **1.020 (1.006-1.034)** | **1.06** | **1.10** | 1.009 (0.990-1.028) | 1.03 | 1.05 | 1.012 (0.991-1.033) | 1.04 | 1.06 | **1.037 (1.012-1.062)** | **1.12** | **1.20** |
| PRAQ-R2 F1 | 24 | **1.074 (1.038-1.112)** | **1.24** | **1.43** | 1.044 (0.996-1.095) | 1.14 | 1.24 | 1.006 (0.955-1.059) | 1.02 | 1.03 | **1.138 (1.074-1.206)** | **1.47** | **1.91** |
|  | 34 | **1.097 (1.058-1.137)** | **1.32** | **1.59** | 1.009 (0.960-1.061) | 1.03 | 1.05 | 1.035 (0.982-1.091) | 1.11 | 1.19 | **1.130 (1.062-1.203)** | **1.44** | **1.84** |
| EPDS  SCL-90 | 24  34  24 | 0.997 (0.977-1.019)  1.011 (0.989-1.034)  0.985 (0.965-1.006) | 0.99  1.03  0.96 | 0.99  1.06  0.93 | 0.982 (0.951-1.014)  0.996 (0.964-1.028)  0.978 (0.948-1.009) | 0.95  0.99  0.94 | 0.91  0.98  0.89 | 1.001 (0.968-1.034)  1.010 (0.976-1.045)  0.998 (0.966-1.031) | 1.00  1.03  0.99 | 1.01  1.05  0.99 | 1.018 (0.980-1.057)  1.037 (0.997-1.078)  1.028 (0.992-1.065) | 1.05  1.12  1.09 | 1.09  1.20  1.15 |
|  | 34 | 1.010 (0.987-1.034) | 1.03 | 1.05 | 1.014 (0.981-1.047) | 1.04 | 1.07 | 1.019 (0.984-1.054) | 1.06 | 1.10 | 1.064 (1.027-1.103) | 1.20 | 1.36 |

**The effect of a rise on questionnaire scores on the risk of selected outcomes as ORs calculated using a 3-point and 5-point rise*

*Table 10.* The association between psychological distress and labor duration, with 3- and 5-point increased OR’s (*significant OR’s bolded)^a^*

| c |  | Time point | Duration of First stage of labor (cervical dilatation) |  |  | Duration of Second stage of labor (active pushing) |  |  |
| --- | --- | --- | --- | --- | --- | --- | --- | --- |
|  |  | (gwk) | Ratio (95% CI) | OR^3^ | OR^5^ | Ratio (95% CI) | OR^3^ | OR^5^ |
| 1^a^ | PRAQ-R2 | 24 | 1.002 (0.998-1.006) | 1.01 | 1.01 | **1.006 (1.001-1.012)** | **1.02** | **1.03** |
|  |  | 34 | 1.000 (0.996-1.005) | 1.00 | 1.00 | 1.004 (0.998-1.009) | 1.01 | 1.02 |
|  |  |  |  |  |  |  |  |  |
|  | PRAQ-R2 F1 | 24 | **1.012 (1.002-1.023)** | **1.04** | **1.06** | **1.033 (1.019-1.047)** | **1.10** | **1.18** |
|  |  | 34 | 1.007 (0.996-1.018) | 1.02 | 1.04 | **1.032 (1.018-1.046)** | **1.10** | **1.17** |
|  |  |  |  |  |  |  |  |  |
|  | EPDS | 24 | 1.004 (0.997-1.010) | 1.01 | 1.02 | 1.005 (0.996-1.013) | 1.02 | 1.03 |
|  |  | 34 | 1.001 (0.995-1.008) | 1.00 | 1.00 | 1.002 (0.993-1.011) | 1.01 | 1.01 |
|  |  |  |  |  |  |  |  |  |
|  | SCL-90 | 24 | 1.000 (0.993-1.006) | 1.00 | 1.00 | 1.005 (0.994-1.013) | 1.02 | 1.03 |
|  |  | 34 | 1.004 (0.997-1.011) | 1.01 | 1.02 | 1.006 (0.997-1.015) | 1.02 | 1.03 |
|  |  |  |  |  |  |  |  |  |
| 2^b^ | PRAQ-R2 | 24 | 1.000 (0.996-1.004) | 1.00 | 1.00 | 1.005 (1.000-1.010) | 1.02 | 1.03 |
|  |  | 34 | 0.998 (0.994-1.002) | 0.99 | 0.99 | 1.003 (0.997-1.008) | 1.01 | 1.02 |
|  |  |  |  |  |  |  |  |  |
|  | PRAQ-R2 F1 | 24 | 1.005 (0.995-1.015) | 1.02 | 1.03 | **1.029 (1.015-1.043)** | **1.09** | **1.15** |
|  |  | 34 | 0.998 (0.988-1.008) | 0.99 | 0.99 | **1.027 (1.014-1.041)** | **1.08** | **1.14** |
|  |  |  |  |  |  |  |  |  |
|  | EPDS | 24 | 1.004 (0.998-1.010) | 1.01 | 1.02 | 1.005 (0.996-1.013) | 1.02 | 1.03 |
|  |  | 34 | 1.000 (0.994-1.006) | 1.00 | 1.00 | 1.001 (0.992-1.010) | 1.00 | 1.00 |
|  |  |  |  |  |  |  |  |  |
|  | SCL-90 | 24 | 1.001 (0.995-1.007) | 1.00 | 1.00 | 1.006 (0.997-1.014) | 1.02 | 1.03 |
|  |  | 34 | 1.002 (0.995-1.009) | 1.01 | 1.01 | 1.005 (0.996-1.014) | 1.02 | 1.03 |

*^a^The effect of a rise on questionnaire scores on the risk of selected outcomes as ORs calculated using a 3-point and 5-point rise*

*Spontaneous vaginal birth, vacuum extraction and ACs included in both analysis*
